# 

**Published:** 1845-09-01

**Authors:** 


BUFFALO

### MEDICAL JOURNAL.

EDITED BY

AUSTIN FLINT, M. D.

**Vol. 1. BUFFALO, SEPTEMBER, 1845. No 4.**

### CONTENTS:

### ORIGINAL COMMUNICATIONS.

Notes of an European Tour. By F. H. HAMILTON, M. D., Prof. of Surgery in Geneva Medical College. P. 73

Report of the Wounded at the Battle of Okee-Chobee, Florida, 25th Dec. 1837. By R. C. WOOD, M. D. Surgeon U. S. Army. - - - 80

Luxations. - - - 85

### EDITORIAL, MEDICAL INTELLIGENCE, &C.

Buffalo Medical Association. Address of the President, JOSIAH TROWBRIDGE, M. D. - - - 88

Account of an Epidemic Fever which occurred at North Boston, Erie County, New-York, during the months of October and November, 1843. By AUSTIN FLINT, M. D., with remarks by a correspondent. - - 91

Elementary and Practical Treatise of Internal Pathology. By GRISOLLE. - - - 95

Protracted Wakefulness. - - - 96

Pthisis Pulmonalis in New Orleans. - - - 96

To Readers and Correspondents. - - - 96

BUFFALO:

C. F. S. THOMAS, PRINTER AND PUBLISHER,

Exchange Buildings, Third Story, Main-Street.

